# Antibiotic repeat prescriptions: are patients not re-filling them properly?

**DOI:** 10.1186/s40545-014-0017-z

**Published:** 2014-12-16

**Authors:** Iman Zayegh, Theresa L Charrois, Jeffery Hughes, Kreshnik Hoti

**Affiliations:** School of Pharmacy, Curtin University, Kent St, Bentley, WA 6102 Australia; Faculty of Pharmacy and Pharmaceutical Sciences, University of Alberta, 116 St and 85 Ave., Edmonton, AB T6G 2R3 Canada

**Keywords:** Antibiotics, Pharmacists, Repeat prescriptions, Australia, Respiratory tract infections

## Abstract

**Objective:**

This study aimed to explore patients’ utilization of repeat prescriptions for antibiotics indicated in upper respiratory tract infections (URTI). An emphasis was placed on whether the current system of repeat prescriptions contributes to patients self-diagnosing infections and if so, identify the common reasons for this.

**Methods:**

This is a prospective study of self-reported use of repeat antibiotic prescriptions by pharmacy consumers presenting with repeat prescriptions for antibiotics commonly indicated in URTIs. Data were collected via self-completed surveys in Perth metropolitan pharmacies.

**Results:**

A total of 123 respondents participated in this study from 19 Perth metropolitan pharmacies. Of the respondents, approximately a third of them (33.9%) presented to the pharmacy to fill their antibiotic repeat prescription one month or more from the time the original prescription was written (i.e. time when original diagnosis was made by a doctor). Over two thirds of respondents indicated to not have consulted their doctor prior to presenting to the pharmacy to have their antibiotic repeat prescription dispensed (i.e. 68.3%). The most common reasons for this were that their ‘doctor had told them to take the second course’ (38%), followed by potential self-diagnosis (29%), i.e. ‘they had the same symptoms as the last time they took the antibiotics’. Approximately one third (33.1%) of respondents indicated they ‘were not told what the repeat prescription was needed for’ when they were originally prescribed the antibiotic. Respondents who presented to fill their repeat prescription more than 2 weeks after the original prescription written were more likely not have consulted their doctor (p = 0.006, 95% CI [1.16, 2.01]) and not to know why their repeat was needed (p = 0.010, 95% CI [1.07, 2.18]).

**Conclusions:**

Findings of this study suggested that the current 12 month validity of antibiotics repeat prescriptions is potentially contributing to patients’ self-diagnosis of URTIs and therefore potential misuse of antibiotics. This may be contributing to the rise of antimicrobial resistance. The study also outlines some common reasons for patients potentially self-diagnosing URTIs when using repeat prescriptions. Larger Australian studies are needed to confirm these findings.

## Introduction

The emergence of antibiotic resistance poses a local and global threat, with significant repercussions for public health [1]. There is a strong evidence linking antibiotic prescribing in primary care with emergence of antibiotic resistance [2]. The widespread and inappropriate use of antibiotics has been accompanied by calls to promote judicious use of antibiotics [1-4].

Amongst developed countries, Australia has one of the highest levels of antibiotic use in the world [5]. Under the Pharmaceutical Benefits Scheme (PBS) specifications, prescribers are permitted to order one repeat supply of antibiotics when the resolution of an infection is unlikely to be achieved with one course [6]. Antibiotic prescriptions are valid for 12 months and repeats can be filled at any time at any pharmacy. The 12 months validity of antibiotic repeat prescriptions in Australia is similar to other therapeutic groups including antihypertensive and lipid modifying agents [6].

Electronic prescribing systems in the Australian general practice setting are often defaulted to automatically generate repeats on prescription medications, including antibiotics [4,7]. The increased number of antibiotic repeats generated by computerized prescribing systems is a potential contributor to the inappropriate use of antibiotics [4,7]. Initiatives have been established to increase prescriber awareness in order to prevent the automatic generation of repeats for antibiotics [7]. Despite these initiatives, it appears that the trend in antibiotic repeat prescribing has not changed significantly and any observed changes in prescribing patterns have been short-lived [8].

There is currently a paucity of literature data surrounding patient utilization of antibiotic repeat prescriptions. It is also not clear what happens in cases when patients delay filling of their antibiotic repeats originally intended for finishing their treatment course following initial diagnosis. Having this in mind, the primary objective of this study was to explore whether the current system of repeat prescribing for antibiotics commonly used in upper respiratory tract infections (URTI) contributes to patients self-diagnosing. Secondary objectives included identifying common reasons for patients self-diagnosing when choosing to use a repeat prescription.

## Methods

Ethics approval was obtained from Curtin University Human Research Ethics Committee.

### Design and setting

This was a prospective study in which data was collected using a questionnaire administered in randomly selected pharmacies in Perth. A list of all Perth Metropolitan pharmacies was generated using the Yellow pages and, using an electronic randomizer (www.randomizer.org), a 20% random sample of pharmacies were selected. Random selection was utilized to maximize representativeness and validity of the study and to allow a wider representation of the population of metropolitan pharmacies in Australia. A total of 67 (i.e. 20% of all pharmacies in the metropolitan area) were invited to take part in the study via telephone calls and posted letters. Pharmacist managers, who were provided with the information surrounding this study, gave verbal consent to take part in the study. The consenting pharmacist provided an information sheet, as well as a self-completed questionnaire to consenting respondents who presented at participating pharmacies with an antibiotic repeat prescription. Data was collected between February and May 2012.

### Questionnaire design

Relevant literature was consulted in designing the questionnaire [9,10] and a focus group session was conducted to assist with its face and content validation. The focus group comprised members of the public who had previously used antibiotic repeats and pharmacists. The repeat prescriptions of interest were those for antibiotics used in URTI. URTI antibiotics were chosen as these are the most commonly dispensed antibiotics [4]. Following consultations with pharmacists in the focus group, we modified the antibiotic list used by Newby et al. [4] and included amoxycillin, amoxycillin with clavulanic acid, cefaclor, roxithromycin, cephalexin and clarithromycin. The first section of the questionnaire collected information on respondents’ demographics and frequency of prescription use. This section was followed by questions about: what the respondent thought that the repeat was being used for; what their original prescription was given for; and whether the respondent had consulted their doctor prior to having the repeat dispensed and, if not, why. Respondents were also asked how long it had been since they had last seen their doctor and if they knew why their doctor had given them the repeat prescription for the antibiotic. The second section of the questionnaire was completed by the pharmacist and the data collected included the date and time of respondents’ presentation, the patient’s concessional status, the date of the original prescription, whether it was computer generated or handwritten prescription, the antibiotic name and dosage form, as well as the number of repeats originally given and the number remaining and post-code of the pharmacy. Pharmacists’ interventions in cases where filling the repeat prescription may have been deemed inappropriate were not recorded as this was outside of the scope of the study.

Throughout the data collection period, participating pharmacies were required to collect data for all consenting eligible respondents (i.e. those presenting with a repeat prescription for antibiotics used in URTI) at any time of the day, seven days a week. This was specified in order to minimize the potential for inconsistencies with data collection between pharmacies and minimize the risk of extraneous variables skewing results, such as certain populations (e.g. elderly patients) presenting at particular times of the day/week compared to other patient groups.

Data collected was entered into SPSS® Vs. 19. Frequency distributions were obtained and data was compared for differences and associations. Relationships between categorical variables were explored using Chi-square test and a p-value of <0.05 was regarded statistically significant. In cases when numbers in cross-tabulation cells were less than five a Fisher’s exact test was used. Confidence intervals were calculated for prevalence estimates.

## Results

A total of 25 pharmacies were recruited out of the 67 contacted pharmacies. Out of 25 pharmacies who initially accepted to participate, six of them did not collect any data. Contacted pharmacies that chose not to take part in the study claimed not to have had the time (41.2%), staff (35.3%) or prescription volume (23.5%) required to participate in the research project.

A total of 123 surveys were completed throughout the collection period. The majority of respondents were female (67%), working full time (41.8%) and with a general concessional PBS status. The majority of respondents were filling a repeat prescription for themselves or their child (84.4%). More detailed demographic data for the respondents are presented in Table 1.

**Table 1:** **Demographic characteristics of patients in the study sample (n = 123)**

**Demographical characteristics**	**n (%)**	**Demographical characteristics**	**n (%)**
Sex		Prescription presented is for:	
Female	67 (54.4)	Myself	94 (77)
Age		My child	9 (7.4)
18-30	29 (23.8)	My partner	19 (15.6)
31-45	28 (23.0)	Educational Status	
46-55	20 (16.4)	Primary School	5 (4.3)
55-65	20 (16.4)	Secondary School	47 (40.2)
65-80	21 (17.2)	University	56 (47.9)
> 80	4 (3.3)	Preferred not to disclose	9 (7.7)
Missing	1	Missing	6
Concessional Status		Employment Status	
General	69 (60.0)	Full-time	51 (41.8)
Concession	36 (31.3)	Part-time	19 (15.6)
Repatriation	4 (3.5)	Casual	14 (11.5)
Number of prescriptions filled for themselves		Retired	29 (23.8)
None	18 (14.6)	Unemployed	9 (7.4)
< 1 per month	48 (39.3)	Missing	1
1 per month	13 (10.7)		
2-5 per month	29 (23.8)		
> 5 per month	14 (11.5)		
Missing	1		

The majority of prescriptions were computer generated (82.6%) and the most common antibiotic prescribed was amoxycillin with clavulanic acid (40.4%) followed by amoxicillin alone (21.1%). The vast majority of antibiotics (88%) were prescribed with one repeat. Of the respondents, 40% of them presented to the pharmacy to fill antibiotic prescription more than 2 weeks after the initial fill date and 33.9% filled their repeat one month or more from the time diagnosis was made. More details about the repeat prescriptions are available in Table 2.

**Table 2:** Patient prescription information (n = 123*)

**Type of information**	**n (%)**	**Type of information**	**n (%)**
Antibiotic		Time between original Rx and repeat filling	
Amoxycillin with clavulanic acid	<2weeks	<2weeks	69 (60)
Amoxycillin	≥2 weeks <1 month	≥2 weeks <1 month	7 (6.1)
Roxithromycin	≥1 month <3 months	≥1 month <3 months	17 (14.8)
Cephalexin	≥3 months <6 months	≥3 months <6 months	9 (7.8)
Clarithromycin	≥6 months	≥6 months	13 (11.3)
Cefaclor	Missing	Missing	8
Missing	14		

*For both categories there were some missing responses.

### Current reasons for using the repeat prescription and original indication of antibiotic prescriptions

Respondents indicated that the most common reasons for which the antibiotic repeats were being used (as perceived by respondents) were ‘cough’ (34.5%), followed by ‘not feeling better after the first course’ (19.0%) ‘earache’ (17.2%) and ‘sore throat’ (16.4%). These were followed by ‘blocked nose’ (12.1%), ‘runny nose’ (9.5%) and ‘starting to get sick and not wanting to get worse’ (7.8%).

The most common reported original indications (as perceived by respondents) for the antibiotic prescriptions were ‘chest infection’ (37.8%), followed by ‘ear infection’ (20.2%), and ‘tonsillitis’ (14.3%). More details in relation to antibiotics original indications as reported by respondents are provided in Table 3.

**Table 3:** **Original indications and prevalence of doctor consultation prior to filling of antibiotic repeat prescriptions (n = 122)**

**Original indication for antibiotic**	**n(%)**	**Doctor consulted about getting the repeat prescription dispensed (within respective original indication)? (YES/NO)**
		**NO; n (%)**
Chest infection	45 (36.9)	33 (73.3)
Ear infection**	24 (19.7)	21 (87.5)
Tonsillitis	17 (13.9)	12 (70.6)
Sinusitis	14 (11.5)	8 (57.1)
‘the flu’*	10 (8.2)	10 (100)
Do not know/remember	4 (3.3)	-
Bronchitis	4 (3.3)	2 (50)
Missing	1	1

*p = 0.030; **p = 0.027.

### Doctor consultation

**Figure 1:**
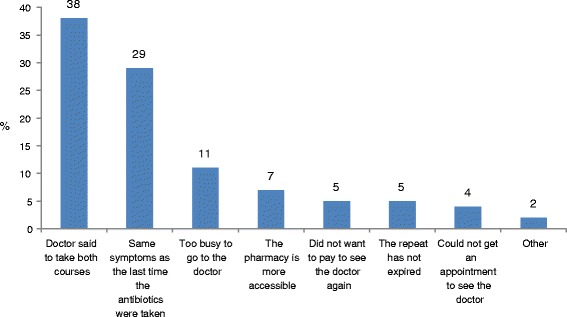
**Reasons patients did not consult their doctor prior to having their repeat dispensed (%).**

The prevalence of respondents that indicated that no doctor consultation was made prior to presenting to the pharmacy to fill their antibiotic repeat prescription was 68.3% (95% CI: 60.0 - 76.6%). Most respondents indicated that they did not consult a doctor prior to having the antibiotic repeat dispensed because their ‘doctor had told them to take the second course’. This was followed by ‘having the same symptoms as the last time they took the antibiotics’. For more details on reasons for having their repeat filled refer to Figure 1. Respondents who presented to fill their antibiotic repeat prescription within 2 weeks of the original prescription being written (i.e. when original diagnosis was made) were significantly more like to have done so based on the consultation with their doctor (p = 0.006, 95% CI [1.16, 2.01]).

A significant association was identified between respondents who reported original indications for ‘the flu’ and ‘ear infection’ and their doctor consultation. In this regard, the majority of respondents did not consult a doctor prior to having the repeat dispensed when the antibiotic was prescribed for ‘the flu’ (100%, Fisher’s exact test: p = 0.030, 95% CI [1.34,1.75] ) or for an ‘ear infection’ (87.5%, Chi-square test: p = 0.027 95% CI [1.12,1.71]. For more details see Table 3.

When comparing respondents age, sex and employment status, no statistical significant differences were seen in rates of doctor consultation prior to their presentation to have the repeat dispensed (p > 0.05). In regards to educational level, respondents with a university level education were more likely not to have consulted a doctor prior to presenting to have the repeat dispensed (Fisher’s exact test: p = 0.019). No significance differences were found between the above demographic factors and whether respondents presented to fill their prescription within 2 weeks of original prescription written (p > 0.05).

### Respondents knowledge regarding use of the repeat

Nearly 40% of respondents indicated they did not to know why the repeat prescription was needed ( i.e. 33.1%) of respondents indicated they were ‘not told by their doctor what the repeat prescription was needed for’ when they were originally prescribed the antibiotics and a further 5.8% responded they ‘could not remember’ why the repeat was given).

A significant association was identified between timing of original prescriptions prescribed and repeat prescriptions dispensed, in relation to respondents’ knowledge regarding use of their repeat. In this regard, respondents who presented to fill the repeat more than 2 weeks after the original prescription was prescribed, were significantly more likely not to know why their repeat was needed (i.e. variables ‘not been told by a doctor what the repeat was needed’ and ‘do not remember’ combined (p = 0.010, 95% CI [1.07, 2.18])).

## Discussion

This study found that the majority of respondents did not consult their doctor prior to presenting to the pharmacy to have their antibiotic repeat dispensed, despite a significant proportion not knowing what the repeat prescription had been provided. Further, 40% of respondents presented to fill the repeat prescription more than two weeks after the original prescription written (1 in 3 patients presented more than one month and 1 in 5 more than 3 months). These observations are consistent with the findings of a Newcastle study conducted in 2003 [4], that suggested a substantial proportion of patients retained their repeats for a considerable amount of time after their original issue. This suggests that antibiotic repeats originally prescribed for URTI are potentially being used for indications other than that what they may have originally been intended by the prescriber, leaving room for patient self-diagnosis.

All respondents who claimed to have been prescribed their antibiotics for ‘the flu’ did not consult their doctor prior to presenting to have the repeat dispensed and compared to other patient-reported indications this was a statistically significant finding. This should be viewed also having in mind that the most common reported reason for respondents presenting to fill the repeats was ‘cough’. The above suggests that in these cases, antibiotic repeat prescriptions could have potentially been used by patients for non-bacterial infections. Antibiotics for URTI are most frequently prescribed antibiotics despite many of such infections being viral in etiology [4,11]. The resulting widespread use of antibiotics is a primary factor in the emergence of antibiotic resistance at both local and regional levels [11].

Approximately a third of respondents indicated their doctor did not tell them why a repeat prescription was needed. This finding suggests potential inadequate communication between the prescriber and patients about their antibiotic use and emphasizes the need for further education focused on addressing this communication gap. This also highlights the significance of pharmacists’ counseling when antibiotic prescriptions are filled, including their repeats. A further exploration of how to better utilize pharmacists skills in improving patients’ knowledge about their repeat prescription should be considered. To assist with this, a better use of dispensing software solutions could also be designed.

It appears that the current system of antibiotic repeat prescriptions where they are valid for 12 months may contribute to patients’ lack of knowledge of the need for their antibiotic repeat prescription. In this regard, respondents who presented to fill their repeat prescriptions ≥ 2 weeks (from the original prescription) were significantly more likely to not know why their repeat was prescribed. It should be noted that current guidelines limit the course of antibiotic use for most URTI for up to 2 weeks [12,13]. The current 12 months repeat validity of antibiotics is currently the same as for prescriptions used in chronic conditions such as hypertension and diabetes despite most bacterial infections being short-term conditions. This leaves room for patients to potentially use their repeats for a condition which is different to the original indication assigned by prescriber and hence engage in self-diagnosis.

This study had a number of important limitations. These limitations firstly pertain to a small sample size. Lack of time and staff were the main reasons for pharmacies failing to recruit more patients. Findings of this study should also be interpreted with caution as some of the key questions were answered based on respondents’ perceived knowledge or awareness of symptoms and current condition. Furthermore, potential recall bias should be recognized, given that respondents may not remember what the doctor had originally told them. In addition, it should be acknowledged that other antibiotics not included in this study are used in URTI, such as erythromycin, azithromycin, doxycycline and dicloxacillin. Our study is also limited by the fact that it did not include rural populations in Australia. This was however done to limit confounding variables such as differing trends in infections and antibiotic use in rural and regional parts of Western Australia. Furthermore, our study was conducted in summer and autumn and there may be seasonal variations in antibiotic use during other times of the year. It should also be noted that in terms of wider interpretation of these findings, not all countries utilize a repeat prescription system for antibiotics.

In spite of these limitations, it is clear that there are issues with the current system of repeats for antibiotic prescriptions especially around their 12 months validity. Given the failure of previous educational campaigns directed to prescribers in order to reduce default repeat generation for antibiotics [7] other measures, such as limiting the time between initial fill and refill on antibiotics, should be considered. Additionally, improving patient education in utilization of repeat prescriptions should also be considered.

## Conclusions

Systems allowing automatic generation of antibiotic repeats at the point of prescribing and repeat validity beyond completion of treatment course have potential to contribute to patients self-diagnosing URTI infections and hence inappropriately using antibiotics. Larger scale studies are needed in order to get a clearer picture of the extent of patient self-diagnosis of URTI infections and its impact on antibiotic resistance.
